# Drivers of Spatial Expansions of Vampire Bat Rabies in Colombia

**DOI:** 10.3390/v14112318

**Published:** 2022-10-22

**Authors:** Zulma E. Rojas-Sereno, Daniel G. Streicker, Andrea Tatiana Medina-Rodríguez, Julio A. Benavides

**Affiliations:** 1 Centro Para la Investigación de la Sustentabilidad y Doctorado en Medicina de la Conservación, Facultad Ciencias de la Vida, Universidad Andres Bello, República 440, Santiago 8320000, Chile; 2 School of Biodiversity, One Health and Veterinary Medicine, University of Glasgow, Glasgow G12 8QQ, UK; 3 Centre for Virus Research, MRC-University of Glasgow, Glasgow G61 1QH, UK; 4 Dirección Técnica de Vigilancia Epidemiológica, Subgerencia de Protección Animal, Instituto Colombiano Agropecuario ICA, Bogotá 110931, Colombia; 5 MIVEGEC, IRD, CNRS, Université de Montpellier, 34394 Montpellier, France

**Keywords:** Latin America, *Desmodus rotundus*, livestock, passive surveillance, spatial epidemiology, zoonosis

## Abstract

Spatial expansions of vampire bat-transmitted rabies (VBR) are increasing the risk of lethal infections in livestock and humans in Latin America. Identifying the drivers of these expansions could improve current approaches to surveillance and prevention. We aimed to identify if VBR spatial expansions are occurring in Colombia and test factors associated with these expansions. We analyzed 2336 VBR outbreaks in livestock reported to the National Animal Health Agency (Instituto Colombiano Agropecuario—ICA) affecting 297 municipalities from 2000–2019. The area affected by VBR changed through time and was correlated to the reported number of outbreaks each year. Consistent with spatial expansions, some municipalities reported VBR outbreaks for the first time each year and nearly half of the estimated infected area in 2010–2019 did not report outbreaks in the previous decade. However, the number of newly infected municipalities decreased between 2000–2019, suggesting decelerating spatial expansions. Municipalities infected later had lower cattle populations and were located further from the local reporting offices of the ICA. Reducing the VBR burden in Colombia requires improving vaccination coverage in both endemic and newly infected areas while improving surveillance capacity in increasingly remote areas with lower cattle populations where rabies is emerging.

## 1. Introduction

Vampire bat-transmitted rabies (VBR) is a lethal zoonosis that represents a public health problem and an economic burden for the livestock sector in Latin America [1,2]. Cattle are particularly affected by VBR and the costs of vaccination and rabies mortality amount to substantial economic losses to small-scale farmers [3]. Since VBR is transmitted by spillover from bats to livestock, outbreaks of VBR usually occur in farming areas with low vaccination coverage of cattle where the virus circulates among vampire bat populations [4,5,6,7]. Improving our understanding of the spatio-temporal dynamics of VBR in bats could support more effective measures to reduce the burden of VBR in livestock; for example, optimizing the spatial or temporal distribution of vaccines to livestock [1,8,9,10].

Spatial expansions of VBR were reported at the range limits of common vampire bats and within their core distribution, generating novel public health and agricultural risks [1,2,11]. For example, in Mexico, VBR has lagged behind the gradual northward expansion of bats towards the United States of America and wave-like expansions into historically rabies-free vampire bat populations were reported in multiple regions of Peru, Argentina, and at a small local scale in Brazil [2,5,12,13,14]. However, the drivers of the occurrence, velocity, or direction of spatial expansions remain unknown but could be affected by environmentally driven changes in the dynamics of rabies within bat populations, influencing bat distribution and mobility across the landscape [1,2,4,5,15,16,17]. For example, differences in cattle density across the landscape could influence the number and size of bat colonies and thus influence viral spread across colonies [15]. An increase in VBR outbreaks was suspected to result from an increase in juvenile dispersal during the wet season in Argentina [18], while bat dispersal is suspected to increase VBR outbreaks during the dry seasons in Colombia [4]. A major challenge is that countries that differ in these environmental conditions also have surveillance systems with differing sensitivity, limiting the value of international comparisons to understand the drivers of the observed variation in VBR spread.

Colombia has reported hundreds of cases of VBR in livestock annually to the Epidemiological Surveillance and Information System of the Instituto Colombiano Agropecuario (ICA) since 1982 [4,19]. The country is composed of five biogeographic regions (Andean, Caribbean, Pacific, Orinoquía, and Amazon), which differ in temperature, precipitation, topography, and livestock density [20,21,22]. For example, the Andean region includes mountains up to 5800mts [21], which may form natural barriers to bat dispersal and therefore VBR propagation. Variable precipitation regimes across regions (e.g., unimodal, bimodal, mixed, and aseasonal) [22], could also influence temporal patterns of bat dispersal and thus VBR spatio-temporal dynamics such as seasonality [4,18]. Since the common vampire bat *Desmodus rotundus* is the main reservoir of rabies in all these biogeographic regions [23], Colombia represents an ideal system to study how different environmental factors could influence VBR spatio-temporal dynamics.

Previous studies of VBR in Colombia found that most outbreaks occurred in areas of high cattle density in the northern region of the Caribbean and the eastern region of Orinoquía [4,6,20,24]. The number of outbreaks in livestock doubled from 2010 to 2019 with three outbreak peaks in 1985, 2010, and 2014 [4,19]. The reasons for these changes in the annual number of outbreaks remain unknown; both spatial expansions and changes in the incidence within endemic areas were hypothesized [2,14,25]. VBR outbreaks were also speculated to be seasonal, with a higher number of outbreaks during the drier months [4]. However, previous studies have not formally tested whether viral expansions are occurring and could explain the observed inter-annual or seasonal changes in VBR incidence. This study aimed to identify whether spatial expansions of VBR are occurring in Colombia and their relationship to the reported burden of rabies, and to test whether biotic or abiotic conditions are associated with viral expansions.

## 2. Materials and Methods

### 2.1. National Surveillance VBR Data

**Figure 1:**
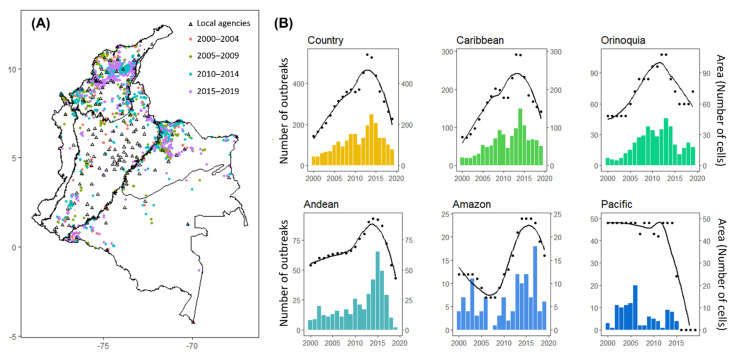
Spatial and temporal distribution of VBR outbreaks in Colombia from 2000 to 2019: (**A**) Locations of VBR outbreaks across the five biogeographic regions of Colombia. Point colors illustrate the five-year periods of outbreak occurrence. Triangles represent the local reporting offices of the ICA’s national rabies surveillance system; (**B**) Bars represent the total number of VBR outbreaks per year. Points correspond to the estimated annual infected area, expressed in number of cells. The number of cells was estimated as the sum of cell grids with a density level higher than the 95th percentile value (i.e., density = 0.01) from a Kernel estimation choosing a bandwidth = 0.01 and a grid = 200 × 200 cells. Lines (black) represent the model prediction tendency estimated using the method ‘loess’ in the geom_smooth function of the ggplot2 in R. Each plot represents estimates at the country or biogeographic region level.

Data on VBR outbreaks were provided by the Epidemiological Surveillance Technical Direction from the ICA, which performs passive surveillance of VBR in livestock. Suspected cases on the basis of neurological signs and mortality are reported by farmers, organizations, veterinary professionals, and other actors related to primary production to specific personnel involved in rabies surveillance at each of the 172 local reporting offices across the country (Figure 1A). Yearly campaigns are carried out to sustain an ‘Early Warning System’ of rabies surveillance in each region by training local stakeholders involved in livestock production to maintain constant surveillance of vampire bat bites and clinical signs compatible with rabies, and to engage in timely reporting of suspected rabies cases to local authorities [19]. In this study, a VBR outbreak was operationally defined when at least one animal from a cluster of animals presenting clinical signs compatible with rabies was confirmed to have died from rabies using the direct fluorescent antibody test [26]. The farm of origin was considered the epidemiological unit for each outbreak. At least 1 veterinarian in charge of rabies surveillance was present in each local reporting office for field assistance during the study period, supervised by thirteen regional epidemiologists, and two national coordinators of rabies surveillance and disease control. No major changes in the number of personnel or reporting offices were observed during our study period. After suspected outbreaks reports were entered into the Official Control Disease National Information System (SINECO), laboratory confirmation and information validation were monitored at the national level [19]. A total of 2336 laboratory-confirmed VBR outbreaks across Colombia occurring between 2000 and 2019 were analyzed. Information on the department, municipality, village, and livestock species was obtained for all outbreaks. GPS coordinates were available for outbreaks occurring from 2010 to 2019. Outbreaks occurring from 2000 to 2009 (856 outbreaks, 37%) had no GPS coordinates recorded and were assigned to the centroid of the nearest village where the farm was located using QGIS 3.3.4 [27].

### 2.2. Colombian Biogeographical Regions and Municipality Data

The five biogeographical regions in Colombia are separated by Andean mountains and include the (I) Caribbean region (dry forest and tropical desert), (II) Pacific region (tropical rainforest), (III) Andean Region (low and high elevation tropical forest), (IV) Amazon region (tropical rainforest), and (V) Orinoquía region (dry tropical grass plains) [21,28]. Each municipality was assigned to a biogeographical region using polygons delimiting each region obtained from the administrative and biogeographic open access shapefiles at the Environmental Information System from Colombia—SIAC (www.siac.gov.co/, accessed on 29 June 2020). Altitude, mean annual precipitation and mean annual temperature raster files of Colombia with a resolution of 0.8 km^2^ were obtained from Bioclim (https://www.worldclim.org/data/bioclim.html, accessed on 29 June 2020) and imported to QGIS 3.3.4. The Point Sampling Tool plugin was used to assign regional and environmental variables values to each outbreak. Addresses of local reporting offices of ICA were obtained from the ICA website (https://www.ica.gov.co/, accessed on 29 June 2020). Annual livestock (horses, pigs, sheep, goats, and buffalos) populations for each municipality were only available from ICA from 2006 to 2019. To avoid excluding outbreaks from 2000 to 2005 in the analysis, the livestock population estimated in 2006 was assigned to those years (2000 to 2005) for each municipality. However, the livestock population remained relatively constant from 2006 to 2017 and only increased after 2017 (Figure S1), so we expect that this extrapolation would not considerably affect the robustness of our results.

### 2.3. Identifying Changes in the Area Affected by VBR

Changes in the infected area, i.e., differences in the geographic extent of locations reporting VBR outbreaks over time, were evaluated using two approaches. First, we calculated the annual number of municipalities that reported VBR for the first time, referred to as ‘new municipalities’ [2]. The area of each municipality was estimated using the areaPolygon function from the Geosphere package in R. Second, we used GPS locations of each VBR outbreak to approximate the annual and monthly infected area. Specifically, we estimated VBR-infected areas at the national and regional levels using kernel density estimation using the bkde2D function from the KernSmooth package in R [29]. We chose a bandwidth = 0.01 (10 km^2^ radius) and a grid = 200 × 200 cells that generated 10 km^2^ cells, an area compatible with the distance of bat movements previously reported [30]. The VBR infected area was estimated as the sum of cell grids with a density level higher than the 95th percentile value (i.e., density = 0.01) and was expressed as a number of infected cells. To verify that the conclusions of our analyses regarding newly infected areas remained similar when the estimated area affected around a location was larger, we also estimated the infected area using a less restricted bandwidth = 0.1 that generates a 100 km^2^ radius and a density level higher than the 88th percentile value (i.e., density = 0.005). We tested the correlation between the annual number of outbreaks and the estimated infected area using a Spearman’s correlation test with the cor.test function in R. Annual changes in the infected area were estimated by comparing the infected area in a specific year to the infected area of the previous year. Large-scale changes in the infected area over time were assessed by comparing the infected area in the second decade of the study (2010–2019) to the infected area in the first decade (2000–2009). ‘New infected areas’ were considered as cells infected in the second period that were not infected in the first period, whereas cells infected in both periods were considered ‘endemic areas’.

### 2.4. Seasonality Analysis

Generalized Additive Models (GAMs) were developed using the gam function in R to test the non-linear annual and monthly variation in the number of outbreaks, the kernel density estimates of the infected area at the national and regional levels, and the annual number of new municipalities reporting outbreaks. GAMs included month (ks = 12 and bs = “cc”) and year (k = 20 and bs = “ps”) as smoothed variables, where k (knots) = temporal dimension used for the spline function and bs = the spline basis, using a cyclic cubic regression (cc) for month and a *p*-spline (ps) for year.

### 2.5. Identifying Drivers of Potential Spatial Expansions

For each new municipality infected, we recorded the ‘time to first outbreak’, referring here to the number of years between the start of our dataset (2000) and the year of the first outbreak reported in that municipality. We then tested whether environmental and anthropogenic drivers could explain the time to the first outbreak using a multivariate regression model. The model was composed of local environmental factors including the estimated cattle population at the year of the first outbreak (or the total livestock population in a separate model) within each municipality, the first outbreak’s altitude, and mean annual precipitation. Since the temperature was highly correlated to altitude (Spearman’s correlation test: Rho = −0.89, *p*-value ≤ 0.01), the regression model only included altitude and precipitation as environmental variables. Since VBR outbreaks are often underreported in Latin America and under-reporting can be correlated with distance to the reporting office [3], we also included distances from the first outbreak in each municipality to the closest reporting office, calculated using the geodist function in R. Given the count nature of the response variable (i.e., years ranging from 0 to 19) and since data were overdispersed (dispersion value = 4.06, *p*-value ≤ 0.01, DHARMa nonparametric dispersion test in R), we built a quasi-Poisson Generalized Linear Mixed Model using a Penalized Quasi-Likelihood with the function glmmPQL in R, including the biogeographical region as a random effect.

To identify whether spatial expansions occurred in a ‘wave-like’ spread where the time of arrival to a municipality increases with its distance to the first case, we intended to include in the model the distance between the first outbreak in each municipality and the first outbreak reported in each biogeographic region [2]. However, the presence of multiple, geographically distant outbreaks in the first month of our dataset suggested that no reliable origin for a ‘wave-like’ spread could be identified, particularly in regions where outbreaks likely occurred prior to 2000 (e.g., the Caribbean). Thus, this variable was ultimately excluded from the analysis.

## 3. Results

### 3.1. Spatio-Temporal Distribution of VBR

From a total of 2336 VBR outbreaks, most were reported in cattle (2037 outbreaks, 87%), followed by horses (285 outbreaks, 12%), pigs (5 outbreaks, 0.2%), sheep (4 outbreaks, 0.2%), goats (2 outbreaks, 0.1%), and buffalos (2 outbreaks, 0.1%). VBR outbreaks were reported in all five biogeographic regions of the country (Figure 1A and Figure S2A) including 27% (297 out of 1123) of all municipalities (Figure S2B). Half of the VBR outbreaks were reported in the Caribbean region (1243 outbreaks, 53%, 132 out of the 297 municipalities reporting outbreaks). The Orinoquía (437 outbreaks, 19%, 34 municipalities) and Andean (421, 18%, 113 municipalities) regions reported a similar number of outbreaks. Outbreaks were only occasionally reported in the Amazon (126, 5%, 31 municipalities) and Pacific (109, 5%, nine municipalities) regions.

The number of VBR outbreaks varied significantly across years and months at the national level (GAM: year effect estimate: edf = 10.25, F = 21.45, *p*-value ≤ 0.01; month effect estimate: edf = 2.66, F = 0.73, *p*-value = 0.03, Table S1), with a peak in 2014 (Figure 1B) and the month of August (Figure S3). However, outbreaks peaked at different years in each biogeographic region and no seasonality was detected when separate models were built for each region (Table S2). There was no correlation between the annual number of VBR outbreaks and the annual total number of livestock (Spearman’s correlation: rho = 0.02, *p*-value = 0.25) between 2006 and 2019. The Pacific was the only region not reporting outbreaks after 2015. The national annual number of outbreaks was strongly correlated with the VBR infected area estimated by Kernel densities (Spearman’s correlation using 10 km^2^ radius: rho = 0.93, *p*-value ≤ 0.01, Spearman’s correlation using 100 km^2^ radius: rho = 0.90, *p*-value ≤ 0.01, Figure 1B), suggesting that the annual burden of VBR may be more tightly linked to the spatial extent of the virus than to local variation in incidence.

### 3.2. Changes in the VBR-Infected Area

**Figure 2:**
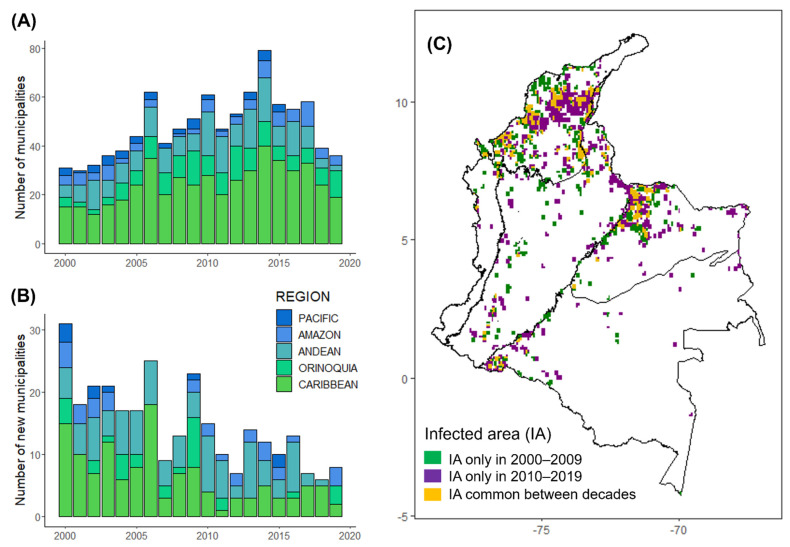
Annual number of municipalities and infected areas in Colombia from 2000 to 2019: (**A**) Bars represent the annual number of municipalities reporting VBR outbreaks; (**B**) Bars represent the annual number of ‘new municipalities’ reporting VBR outbreaks for the first time. Each bar division illustrates a biogeographic region; (**C**) Comparison between the infected area (IA) in the second (2010–2019) and the first (2000–2009) decade of the study. ‘New infected areas’ were considered as cells reporting VBR outbreaks in the second decade that did not report outbreaks in the first decade (cells in purple), whereas cells reporting outbreaks in both decades were considered as ‘endemic areas’ (cells in yellow). The infected area was estimated using a bandwidth = 0.01 that generated a 10 km^2^ radius.

From a total of 297 municipalities reporting VBR outbreaks between 2000 and 2019, the annual number of infected municipalities ranged from 30 to 72 (mean ± SD = 47 ± 12). The highest number of municipalities reporting outbreaks coincided with a peak in the number of outbreaks in 2014 (72 municipalities and 247 outbreaks), when all regions reported outbreaks (Figure 2A). An average of 15 municipalities [SD = 7, range: 6–31] reported VBR outbreaks for the first time each year. Strikingly, these ‘new municipalities’ accounted for 24% (554 outbreaks) of all outbreaks and an average of 27% [SD = 18, range: 10−69] of outbreaks per year. Additionally, an average of 38% [SD = 15, range: 10–58] of the area infected per year was reported among ‘new municipalities’, implying that a considerable portion of the rabies burden occurs in areas that would have been considered rabies-free the prior year. Although new municipalities were reported throughout the study, we noted a marked decrease in the rate that new municipalities were reporting VBR over time (GAM: edf = 1.00, F = 9.49, *p*-value ≤ 0.01, Table S3; Figure 2B). Within biogeographic regions, the number of new municipalities significantly decreased in the Caribbean (Caribbean GAM: edf = 1.71, F = 5.24, *p*-value = 0.01, Table S4, Figure S4) but not significantly in other regions when considered alone.

As measured by kernel densities, the VBR-infected area varied significantly across years and months (GAM: year effect estimate: edf = 8.75, F = 18.23, *p*-value ≤ 0.01; month effect estimate: edf = 1.95, F = 0.63, *p*-value = 0.02, Table S5), with different peaks in each region (e.g., Caribbean, Orinoquía, Andean and Pacific) (Figure 1B and Figure S5). However, the Caribbean was the only region with a significant monthly variation (Caribbean GAM: edf = 1.82, F = 0.54, *p*-value = 0.03, Table S6), with a peak in the infected area during June (Figure S5). An average of 57% (SD = 16, ranging between 30–88 using a 10 km^2^ radius) or 31% (SD = 12, ranging between 14–59 using a 100 km^2^ radius) of the annual area infected across the country originated from new cells reporting VBR outbreaks. Comparing the infected area between 2000–2009 and 2010–2019, 49% (1025 out of 2083 infected cells using a 10 km^2^ radius) or 29% (1374 out of 4716 infected cells using a 100 km^2^ radius) of the total area infected only reported outbreaks during the second decade (Figure 2C, Figure S6 and Figure S7). Cells infected for the first time in the second decade were also identified in municipalities previously infected during the first decade (Figure S7).

### 3.3. Potential Drivers of Spatial Expansions

**Figure 3:**
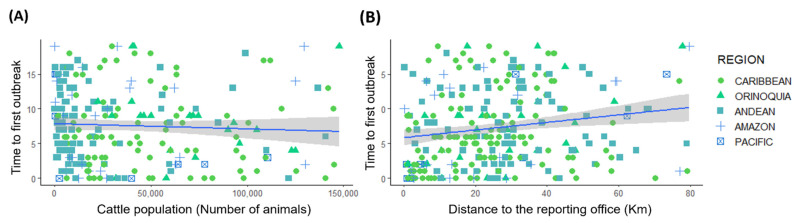
Influence of significant biotic and abiotic conditions on the time of arrival of the first VBR outbreak at each municipality: (**A**) Correlation between the time of the first outbreak (e.g., number of years since 2000) in a municipality and its cattle population. Total livestock population was tested in a different model instead of ‘number of cattle’ without statistical significance (glmmPQL: Estimate = −1.2 × 10^−6^, *p*-value = 0.12). Each point shape represents a biogeographical region; (**B**) Correlation between the time of the first outbreak in a municipality and the geographical distance of that outbreak to the closest reporting office. Lines (blue) represent the tendency estimated for the model and the shadow represents the confidence interval (CI = 95%, grey) estimated using the method ‘lm’ in the geom_smooth function ggplot2 in R.

The time to the first outbreak in each municipality was negatively correlated with the municipality’s number of cattle (glmmPQL, Estimate = −2.0 × 10^−6^, *p*-value = 0.04, Figure 3A) and was positively correlated with the distance from the outbreak to the reporting office (glmmPQL: Estimate = 2.34 × 10^−3^, *p*-value = 0.01, Table 1, Figure 3B and Figure S8). In contrast, the time to the first outbreak was not significantly correlated with altitude, annual precipitation, or the total livestock population (tested in a different model instead of ‘number of cattle’, glmmPQL: Estimate = −1.2 × 10^−6^, *p*-value = 0.12).

## 4. Discussion

Although VBR is considered an endemic disease affecting livestock in Colombia, a poor understanding of its spatio-temporal dynamics limits the effectiveness of measures to anticipate and prevent VBR outbreaks. Our analyses of VBR outbreaks in livestock passively reported to ICA between 2000 and 2019 revealed that temporal changes in VBR-infected areas were consistent with VBR spatial expansions, with more than 30% of the annual infected area originating from newly infected cells and municipalities, and 49% of the infected area in 2010−2019 not reported as infected in the previous decade. Spatial expansions of VBR in Colombia appeared to be decelerating and the virus arrived later in areas with lower cattle populations and located far from local reporting offices. Given our finding that a considerable portion of the national burden of VBR mortality in livestock was attributable to spatial expansions of the virus, our results highlight the need to fortify rabies surveillance and spillover prevention at viral range limits.

Over the 20 years of surveillance data that we analyzed, changes in the VBR-infected area were observed at the country and regional levels (Figure 1B). However, no monotonic trend was observed, with peaks in the infected area during different years across different regions. Changes in the area reporting VBR in livestock could be explained by annual changes in the spread of VBR among bat populations, in part due to spatial expansions as previously observed in Brazil, Mexico, and Peru [2,5,14,31]. For example, changes in bat dispersal or behavior due to environmental changes or culling could affect viral transmission between bat colonies [4,32,33]. New lineages of VBR-colonizing bat populations could also generate changes in the spread of VBR and thus in the area reporting outbreaks in livestock [7,25]. Vampire bat distribution could also be expanding in some areas as a consequence of climate change, an increasing number of human-made structures that could be used as roosts (e.g., mines, tunnels, and abandoned houses), and expansion of livestock populations [16,34]. However, previous studies have shown that VBR spatial expansions such as traveling waves occur in areas with already established vampire bat populations rather than by the expansion of vampire bat populations to new areas [2]. Alternatively to changes in rabies circulation among bats or expansion in the bat distribution, annual variation in the area covered by livestock vaccination could also result in changes in the VBR mortality reported [3], with a higher infected area reported during years of lower vaccination coverage. Thus, future research including estimates of vaccination coverage at the municipality level could contribute to a better understanding of what drives the observed changes in the area reporting VBR outbreaks.

Regardless of the mechanism underlying temporal variation in the affected area of Colombia, our analysis suggests that the burden of VBR is tightly linked to the spatial extent of the virus at any point in time. Specifically, we found a close correspondence between estimates of the infected area and the number of outbreaks reported, suggesting that the burden of rabies in livestock is predominately driven by the presence or absence of the virus rather than local variation in incidence within endemically infected vampire bat populations. Biologically, this pattern might arise through the combination of the extremely low incidence of rabies in vampire bat populations (such that variation in levels of viral circulation is trivial) and a high rate of spillover when the virus is circulating locally due to the high contact rates between bats and livestock [35]. If verified, this finding would have important implications for rabies management since interventions that aim to reduce rabies incidence in vampire bat populations, such as bat population control, might be less effective than direct measures at the bat–livestock interface such as livestock vaccination. We also found that a substantial fraction of all outbreaks occurred in previously rabies-free areas, which is surprising given the relative size of these areas compared to where the virus has been historically endemic. This relatively high burden in historically rabies-free areas is likely to be exacerbated by low or absent livestock vaccination in areas that have not historically been affected, highlighting the potential gains from fortifying rabies surveillance and vaccination efforts at viral range limits [3].

Our analyses suggest that spatial expansions of VBR in Colombia are occurring but have decelerated through time. The slowing rate of expansion could reflect the exhaustion of available rabies-free municipalities suited for both vampire bats and cattle. For example, the Caribbean region had already reported VBR outbreaks in half of its municipalities by the end of our study period, although a few new municipalities are still reported annually [1,4,9]. Our inability to infer the geographic origins or routes of viral expansions, as was possible elsewhere in South America [2,13], also supports the conclusion that at the start of our study, VBR had already established within some or all biogeographical regions, with the observed expansions perhaps emanating in non-linear routes or from multiple origins within a region. Despite this complexity, our study was, for the first time, able to identify correlates of the timeline of VBR arrival to locations. We found that spatial expansions arrived later in municipalities with low cattle populations, which we hypothesize may reflect the epidemiological isolation of these areas arising from smaller or more sparsely connected vampire bat populations [15]. It is alternatively possible that areas with low livestock density instead have fewer real or reported spillovers due to the smaller number of susceptible animals; however, a study in Peru found that reporting was negatively (not positively) related to herd size and was strongly driven by the historical presence of VBR, both arguing against effects of surveillance bias alone [3,36]. We also found evidence for delayed arrival (or reporting) of VBR in municipalities far from reporting offices. This result is likely to reflect both the true geographic isolation of these areas concerning VBR spread and lower reporting rates due to logistical constraints, such that VBR may take more time to be detected [2]. Unfortunately, data on reporting efforts at the municipality level were not available for our study, requiring future research to discriminate between these two alternative scenarios. Regardless, increased recognition of VBR in the most isolated areas adds logistical challenges to the national rabies program relying on livestock vaccination campaigns to reduce the VBR burden [3,36].

Instead of resulting from spatial expansions, temporal changes in the VBR-infected area could result from VBR endemic circulation across the country combined with heterogeneous surveillance efforts that increased over time, generating new VBR reports in areas previously considered as VBR-free when surveillance became sufficient to detect an outbreak. Although data on surveillance efforts during our study period are not available for Colombia to test this hypothesis, no changes in surveillance strategy or capacity were reported at such a large scale that would be compatible with an increase in almost half of the area reporting VBR in the second decade of our study period [19,37]. Further, earlier studies argued that data collected through similar passive surveillance systems provide an accurate reflection of viral arrival to new areas suggesting, as described above, that we would have been unlikely to find a negative relationship between the time until VBR arrival and livestock density if observations of outbreaks were based on reporting alone [2,13]. Finally, given that outbreaks in newly infected areas are likely to be larger due to low vaccination coverage, we speculate that such a sudden increase in mortality would be readily reported [38].

Despite the occurrence of spatial expansions of VBR outbreaks in Colombia, around half of annual outbreaks still occur in ‘endemic areas’. Thus, VBR burden reduction will require improving vaccination coverage in both endemic areas and newly infected municipalities/areas. Our findings suggest that vaccination programs in municipalities previously reporting outbreaks were insufficient to eliminate livestock deaths in the following years. Mandatory vaccination in high-risk zones was established by the ICA’s animal health program in 2003 but has been firmly implemented since 2015 [39,40].However, vaccination coverage remains limited for restricted laboratory production and other reasons that remain unclear. Thus, identifying factors that limit vaccination would help reduce the VBR burden in endemic areas. For example, farmers’ low knowledge of veterinary public authorities and farms located at a higher elevation, rather than a low socio-economic status, were associated with low rabies vaccination intake in Peru [3]. In contrast, reducing the burden of rabies in newly infected areas, which comprised on average at least 27% of outbreaks per year, requires improved epidemiological capacity to forecast the routes and velocity of viral invasions and how these are affected by environmental conditions. As such, our study is a starting point toward the eventual aim of preventive vaccination in areas with emerging risks.

## 5. Conclusions

Overall, our study suggests that part of the changes in the area reporting VBR in the livestock of Colombia could result from spatial expansions of VBR. Our findings suggest that reducing the VBR burden will require improving vaccination coverage in both endemic areas and newly infected areas. This study supports previous work identifying spatial expansions in Brazil, Mexico, and Peru, and suggests that VBR is slowing in speed across Colombia. Delayed and still ongoing viral detections in municipalities with lower cattle populations and those farthest from the local reporting offices indicate surveillance gaps in remote areas that are likely to decrease outbreak predictability, which may intensify losses in newly infected areas.

## Figures and Tables

**Table 1:** Drivers correlated with the time to the first VBR outbreak appearance in each outbreak location: Results from a quasi-Poisson Generalized Linear Mixed Model using a Penalized Quasi-Likelihood with the glmmPQL function in R, including region as a random effect. Time to the first outbreak was not significantly correlated to the total livestock population tested in a different model instead of ‘number of cattle’ (glmmPQL: Estimate = −1.2 × 10^−6^, *p*-value = 0.12). Asterisks identified values considered as statistically significant at *p*-value < 0.05.

Variable	Value	Std. Error	DF	t-Value	*p*-Value
(Intercept)	1.88	0.15	239	12.39	0.00
Altitude	1.59 × 10^−5^	8.02 × 10^−5^	239	0.20	0.84
Precipitation	5.16 × 10^−5^	5.66 × 10^−5^	239	0.91	0.36
Cattle population	−2.00 × 10^−6^	9.50 × 10^−7^	239	−2.12	0.04 *
Distance to the reporting office	2.34 × 10^−3^	9.43 × 10^−4^	239	2.48	0.01 *

## Data Availability

VBR outbreaks and annual livestock population data were obtained from the Epidemiological Surveillance Technical Direction of the ICA. Data can be publicly requested directly from ICA.

## Supplementary Materials

The following supporting information can be downloaded at: https://www.mdpi.com/article/10.3390/v14112318/s1, Figure S1. Annual number of livestock in Colombia from 2006 to 2019; Table S1: GAM model for seasonality of the number of VBR outbreaks at the national level from 2000 to 2019; Figure S2: Spatial distribution of VBR outbreaks in Colombia from 2000 to 2019; Table S2: GAM model for seasonality of the number of VBR outbreaks at the regional level from 2000 to 2019; Figure S3: Annual and monthly distribution of the number of VBR outbreaks from 2000 to 2019; Table S3: GAM model for seasonality of the number of new municipalities reporting VBR outbreaks for the first time at the national level from 2000 to 2019; Figure S4: Annual distribution of the number of new municipalities reporting VBR outbreaks for the first time at the national level from 2000 to 2019; Table S4: GAM model for seasonality of the new municipalities reporting VBR outbreaks for the first time at the regional level from 2000 to 2019; Figure S5: Annual and monthly distribution of the infected area covered from 2000 to 2019; Table S5: GAM model for seasonality of the VBR infected area covered at the national level from 2000 to 2019; Figure S6: Comparison between the infected area in the second decade of the study (2010–2019) and in the first decade (2000–2009); Table S6: GAM model for seasonality of the VBR infected area covered at the regional level from 2000 to 2019; Figure S7: Spatial distribution of the first VBR outbreak occurrence in Colombia from 2000 to 2019; Figure S8: Drivers correlated to the time of arrival of the first VBR outbreak at a municipality according to biogeographic region.
