# Supplementary material for: Drivers of Spatial Expansions of Vampire Bat Rabies in Colombia
Source: Viruses. 2022 Oct 22;14(11):2318. doi: 10.3390/v14112318 (PMC9697077; doi:10.3390/v14112318)
Supplement: Supplementary file 1 [file viruses-14-02318-s001.zip › viruses-1954616-supplementary.pdf]

Supporting Information

Table S1. GAM model for seasonality of the number of VBR outbreaks at the national level from 2000 to 2019.

Model country: s(Month) + s(Year)

	**Term**	**Estimate**	**Std. Error**	**t-value**	**p-value**
**Parametric coefficients**	Intercept	9.73	0.27	36.52	<0.01
	**Term**	**EDF**	**Ref.df**	**F**	**p-value**
**smoothed terms**	Month	2.66	10.00	0.73	0.03*
	Year	10.25	12.05	21.45	<0.01*

Results for a Generalized Additive Model the *gamm* function in R. Asterisk identified values statistically significant at p-value <0.05.

Table S2. GAM model for seasonality of the number of VBR outbreaks at the regional level from 2000 to 2019.

Model by region: s(Month) + s(Year)

**Region**		**Term**	**Estimate**	**Std. Error**	**t-value**	**p-value**
**Caribbean**	**Parametric coefficients**	Intercept	5.18	0.19	28.01	<0.01
		**Term**	**EDF**	**Ref.df**	**F**	**p-value**
	**smoothed terms**	Month	1.4	10.0	0.31	0.09
		Year	10.1	11.89	15.84	<0.01*
		**Term**	**Estimate**	**Std. Error**	**t-value**	**p-value**
**Orinoquía**	**Parametric coefficients**	Intercept	1.82	0.10	18.34	<0.01
		**Term**	**EDF**	**Ref.df**	**F**	**p-value**
	**smoothed terms**	Month	2.53×10^{-4}	10.0	0.00	0.98
		Year	4.99	6.12	12.92	<0.01*
		**Term**	**Estimate**	**Std. Error**	**t-value**	**p-value**
**Andean**	**Parametric coefficients**	Intercept	1.75	0.11	16.76	<0.01

		Term	EDF	Ref.df	F	p-value
	**smoothed terms**	Month	6.20×10^{-4}	10.0	0.00	0.593
		Year	8.67	10.74	11.97	<0.01*
		**Term**	**Estimate**	**Std. Error**	**t-value**	**p-value**
**Amazon**	**Parametric coefficients**	Intercept	0.53	0.06	8.37	<0.01
		**Term**	**EDF**	**Ref.df**	**F**	**p-value**
	**smoothed terms**	Month	0.86	10.0	0.12	0.24
		Year	3.30	4.12	2.48	0.04*
		**Term**	**Estimate**	**Std. Error**	**t-value**	**p-value**
**Pacific**	**Parametric coefficients**	Intercept	0.45	0.06	7.39	<0.01
		**Term**	**EDF**	**Ref.df**	**F**	**p-value**
	**smoothed terms**	Month	5.03×10^{-4}	10.0	0.00	0.54
		Year	4.73	5.89	4.37	<0.01*

Results for a Generalized Additive Model the *gamm* function in R. Asterisk identified values statistically significant at p-value <0.05.

Table S3. GAM model for seasonality of the number of new municipalities reporting VBR outbreaks for the first time at the national level from 2000 to 2019.

Model country: s(Month) + s(Year)

	Term	Estimate	Std. Error	t-value	p-value
**Parametric coefficients**	Intercept	1.81	0.08	22.08	<0.01

	Term	EDF	Ref.df	F	p-value
**smoothed terms**	Month	3.19×10^{-4}	10.00	0.00	0.52
	Year	1.00	1.00	9.49	<0.01*

Results for a Generalized Additive Model the *gamm* function in R. Asterisk identified values statistically significant at p-value <0.05.

Table S4. GAM model for seasonality of the new municipalities reporting VBR outbreaks for the first time at the regional level from 2000 to 2019.

Model by region: s(Month) + s(Year)

Region		Term	Estimate	Std. Error	t-value	p-value
	**Parametric coefficients**	Intercept	0.78	0.06	12.81	<0.01
**Caribbean**		**Term**	**EDF**	**Ref.df**	**F**	**p-value**
	**smoothed terms**	Month	0.63	10.0	0.08	0.27
		Year	1.71	2.12	5.24	0.01*
		**Term**	**Estimate**	**Std. Error**	**t-value**	**p-value**
	**Parametric coefficients**	Intercept	0.18	0.04	5.09	<0.01
**Orinoquía**		**Term**	**EDF**	**Ref.df**	**F**	**p-value**
	**smoothed terms**	Month	3.01×10^{-4}	10.0	0.00	0.51
		Year	1.00	1.00	0.66	0.42
		**Term**	**Estimate**	**Std. Error**	**t-value**	**p-value**
	**Parametric coefficients**	Intercept	0.60	0.06	10.53	<0.01
**Andean**		**Term**	**EDF**	**Ref.df**	**F**	**p-value**
	**smoothed terms**	Month	1.14×10^{-4}	10.0	0.00	0.82
		Year	2.25	2.81	1.00	0.32
		**Term**	**Estimate**	**Std. Error**	**t-value**	**p-value**
	**Parametric coefficients**	Intercept	0.19	0.03	6.14	<0.01
**Amazon**		**Term**	**EDF**	**Ref.df**	**F**	**p-value**
	**smoothed terms**	Month	0.95	10.0	0.15	0.19
		Year	3.53	4.40	2.39	0.05
		**Term**	**Estimate**	**Std. Error**	**t-value**	**p-value**
	**Parametric coefficients**	Intercept	0.06	0.02	2.82	0.01
**Pacific**		**Term**	**EDF**	**Ref.df**	**F**	**p-value**
	**smoothed terms**	Month	0.70	10.0	0.1	0.24
		Year	2.23	2.79	2.26	0.13

Results for a Generalized Additive Model the *gamm* function in R. Asterisk identified values statistically significant at p-value <0.05.

Table S5. GAM model for seasonality of the VBR infected area covered at the national level from 2000 to 2019.

Model 1: s(Month) + s(Year)

		Term	Estimate	Std. Error	t-value	p-value
	**Parametric coefficients**	Intercept	27.15	0.68	39.94	<0.01
**Country**		**Term**	**EDF**	**Ref.df**	**F**	**p-value**
	**smoothed terms**	Month	1.95	10.00	0.63	0.02*
		Year	8.75	10.43	18.93	<0.01*

Results for a Generalized Additive Model the *gamm* function in R. Asterisk identified values statistically significant at p-value <0.05.

Table S6. GAM model for seasonality of the VBR infected area covered at the regional level from 2000 to 2019.

Model by region: s(Month) + s(Year)

Region		Term	Estimate	Std. Error	t-value	p-value
	**Parametric coefficients**	Intercept	14.55	0.42	34.96	<0.01
**Caribbean**		**Term**	**EDF**	**Ref.df**	**F**	**p-value**
	**smoothed terms**	Month	1.82	10.00	0.54	0.03*
		Year	9.19	10.91	15.42	<0.01*
		**Term**	**Estimate**	**Std. Error**	**t-value**	**p-value**
	**Parametric coefficients**	Intercept	6.38	0.21	30.94	<0.01
**Orinoquía**		**Term**	**EDF**	**Ref.df**	**F**	**p-value**
	**smoothed terms**	Month	7.64×10^{-4}	10.00	0.00	0.85
		Year	6.78	8.21	9.42	<0.01*
		**Term**	**Estimate**	**Std. Error**	**t-value**	**p-value**
	**Parametric coefficients**	Intercept	6.22	0.22	28.23	<0.01
**Andean**		**Term**	**EDF**	**Ref.df**	**F**	**p-value**
	**smoothed terms**	Month	1.47	10.00	0.37	0.06
		Year	4.98	6.11	4.92	<0.01*
		**Term**	**Estimate**	**Std. Error**	**t-value**	**p-value**
	**Parametric coefficients**	Intercept	1.67	0.17	9.83	<0.01
**Amazon**		**Term**	**EDF**	**Ref.df**	**F**	**p-value**
	**smoothed terms**	Month	1.61	10.00	0.35	0.09
		Year	3.36	4.16	2.28	0.05
		**Term**	**Estimate**	**Std. Error**	**t-value**	**p-value**

		Term	EDF	Ref.df	F	p-value
	**Parametric coefficients**	Intercept	4.22	0.06	72.61	<0.01
**Pacific**		**Term**	**EDF**	**Ref.df**	**F**	**p-value**
	**smoothed terms**	Month	0.92	10.00	0.15	0.18
		Year	11.76	13.60	54.94	<0.01*

Results for a Generalized Additive Model the *gamm* function in R. Asterisk identified values statistically significant at p-value <0.05.

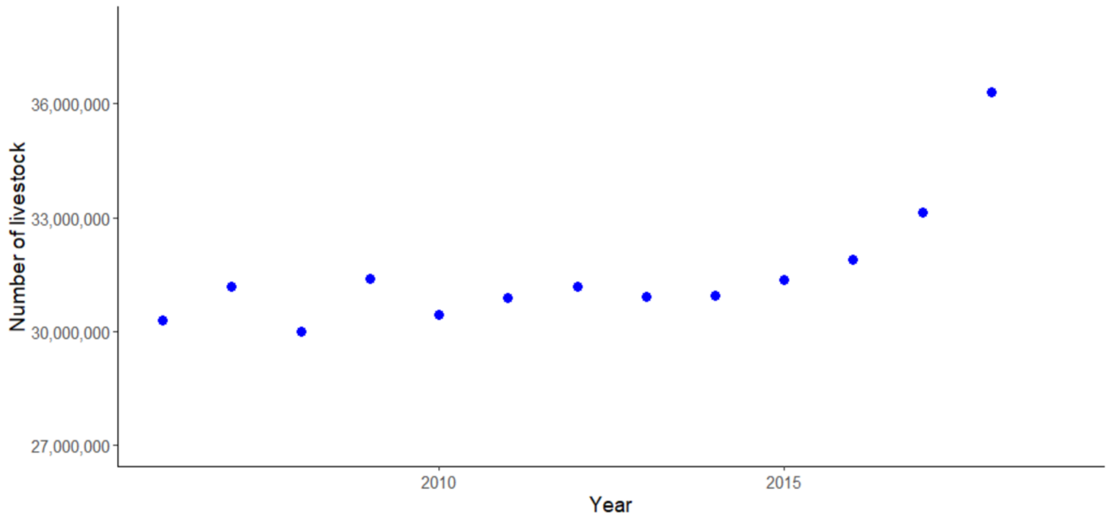

Figure S1. Annual number of livestock population in Colombia from 2006 to 2019.

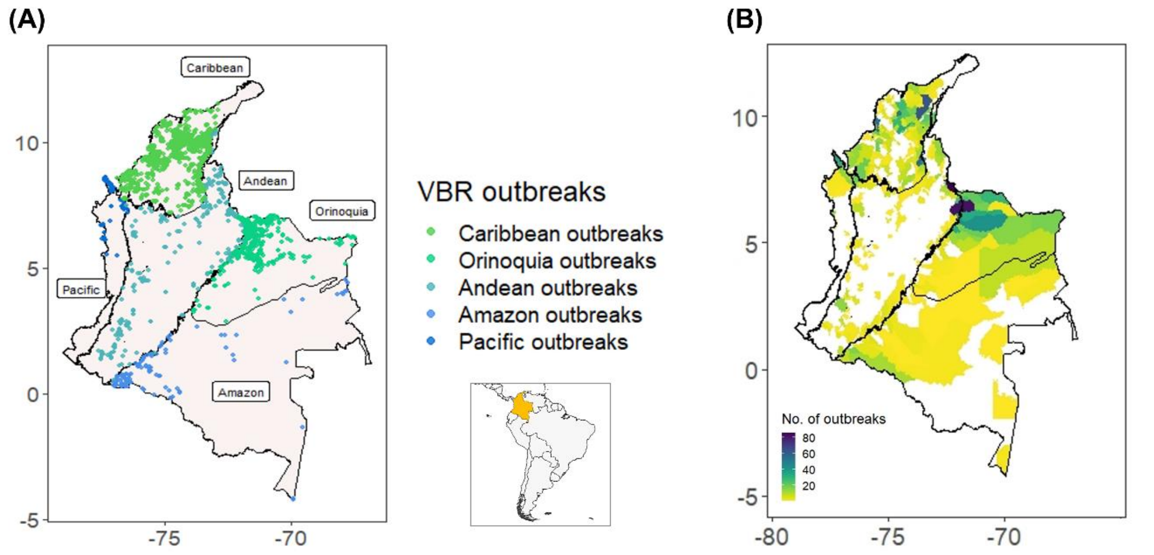

Figure S2. Spatial distribution of VBR outbreaks in Colombia from 2000 to 2019: (**A**) Locations of VBR outbreaks across Colombian five biogeographic regions; (**B**) Number of VBR outbreaks by municipality from 2000 to 2019. Darker colors (blue) represent a higher number of outbreaks reported in a municipality.

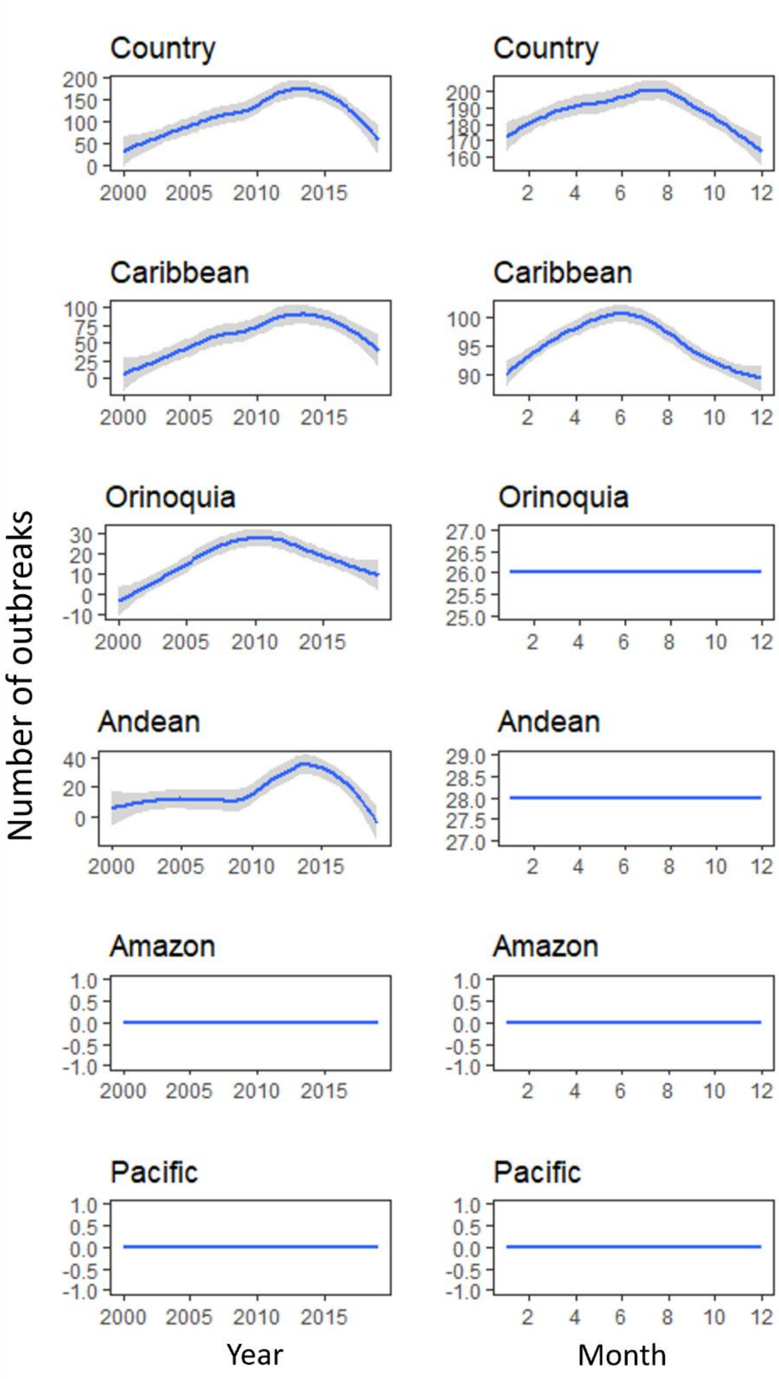

Figure S3. Annual and monthly distribution of the number of VBR outbreaks from 2000 to 2019: Lines (blue) represent the tendency estimated for the prediction of the model and the shadow represents the confidence interval (CI=95%, grey) estimated using the method 'loess' in the *geom_smooth* function in ggplot2 in R. Each plot represents estimates at the country or biogeographic region level. Columns showed the year (right) and month (left) of occurrence.

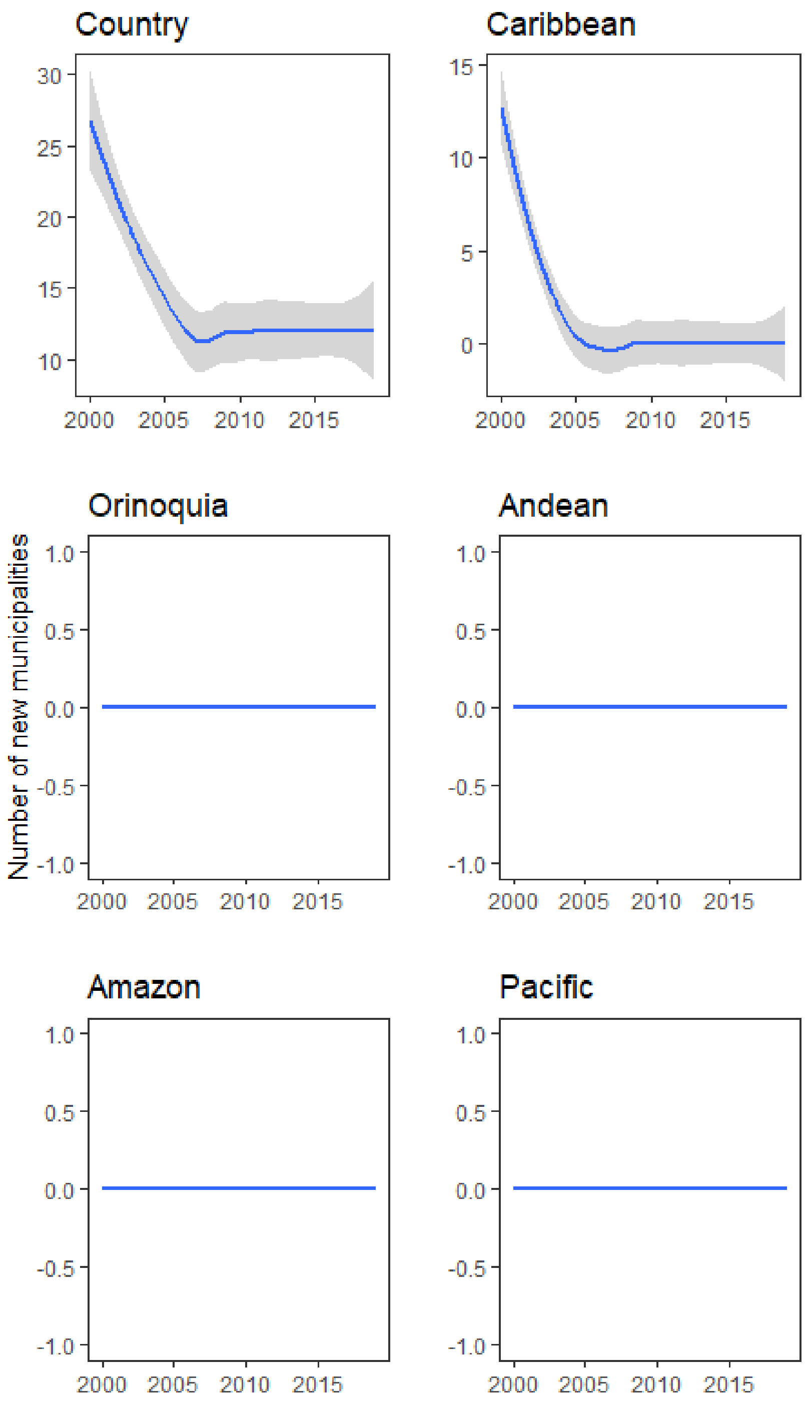

Figure S4. Annual distribution of the number of new municipalities reporting VBR outbreaks for the first time at the national level from 2000 to 2019: Lines (blue) represent the tendency estimated for the prediction of the model and the shadow represents the confidence interval (CI=95%, grey) estimated using the method 'loess' in the *geom_smooth* function in ggplot2 in R. Each plot represents estimates at the country or biogeographic region level.

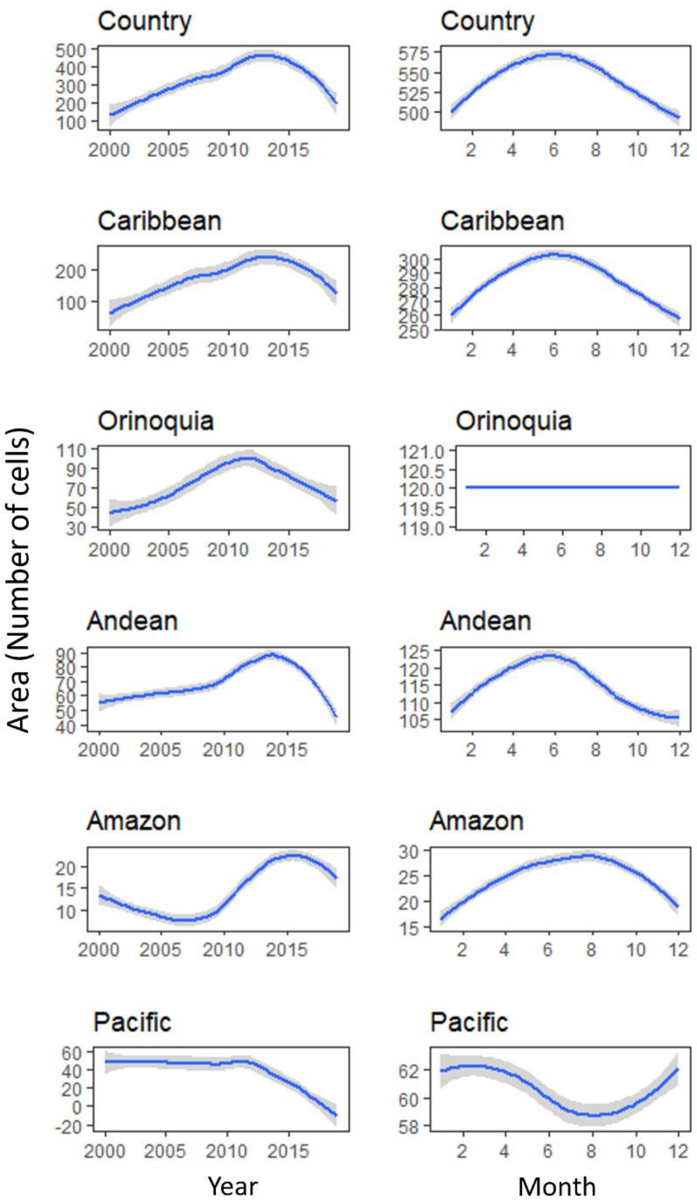

Figure S5. Annual and monthly distribution of the infected area covered from 2000 to 2019: Lines (blue) represent the tendency estimated for the prediction of the model and the shadow represents the confidence interval (CI=95%, grey) estimated using the method 'loess' in the *geom_smooth* function in ggplot2 in R. Each plot represents estimates at the country or biogeographic region level. Columns showed the year (right) and month (left) of occurrence.

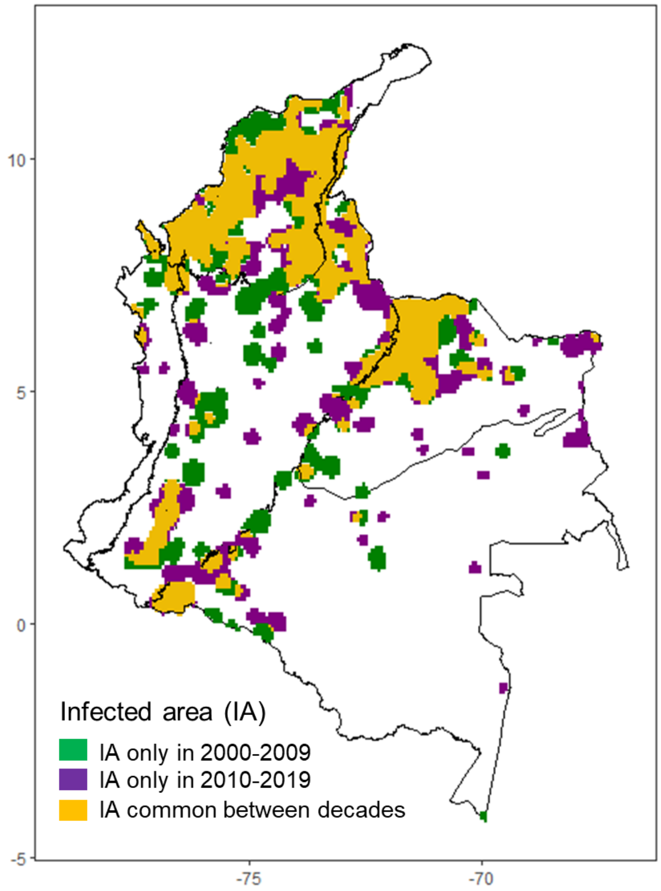

Figure S6. Comparison between the infected area in the second decade of the study (2010-2019) and in the first decade (2000-2009): 'New infected areas' were considered as cells reporting VBR outbreaks in the second decade that did not report outbreaks in the first decade (cells in purple), whereas cells reporting outbreaks in both decades were considered as 'endemic areas' (cells in yellow). Estimation of the infected area estimated used a bandwidth=0.1 that generates a 100 km^2 radius.

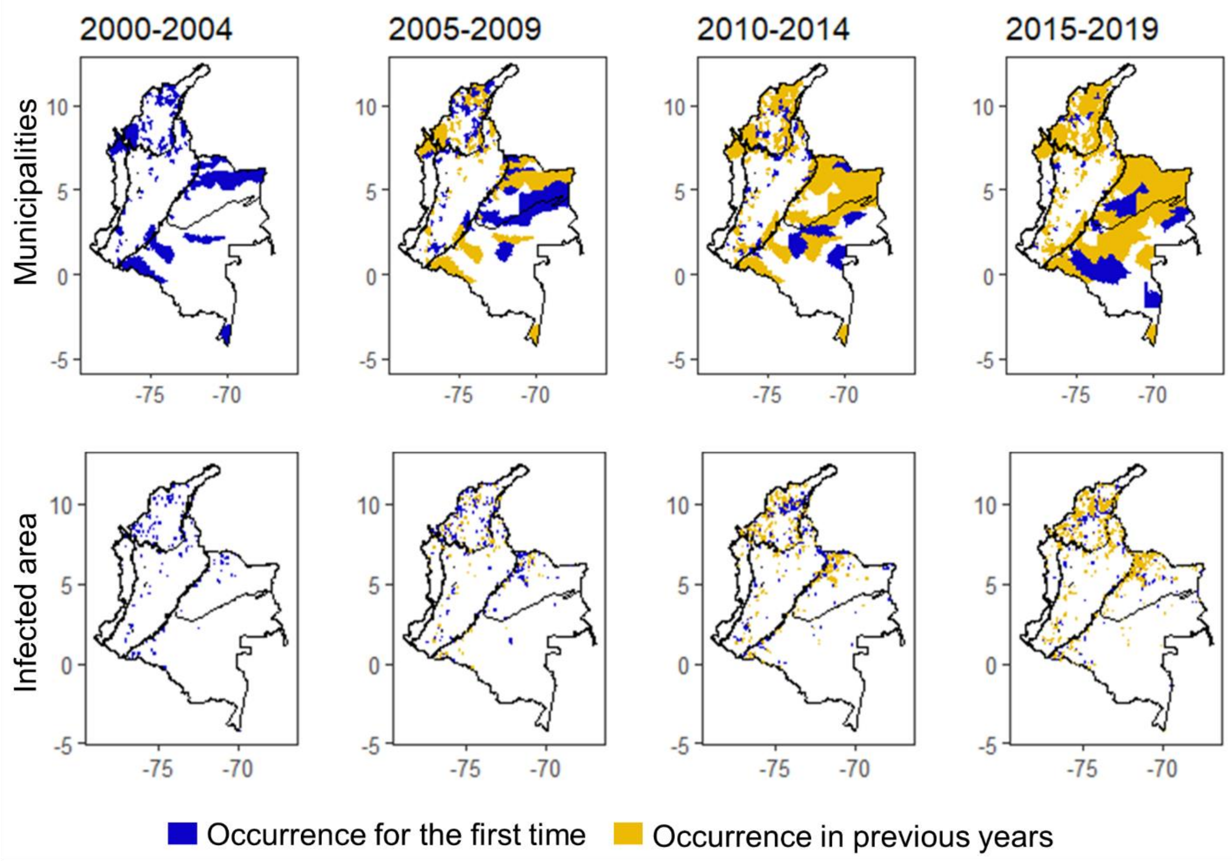

Figure S7. Spatial distribution of the first VBR outbreak occurrence in Colombia from 2000 to 2019: Each plot represents a five-year period of VBR outbreak occurrence by municipalities (top) and infected area (bottom). Colors illustrate VBR outbreak occurrence for the first time (blue) and the occurrence of previous years (yellow).

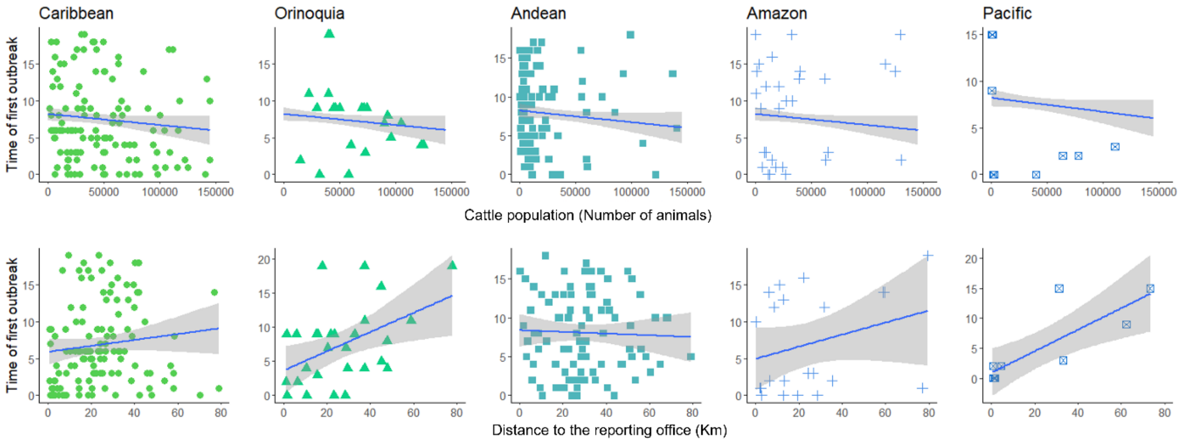

Figure S8. Drivers correlated to the time of arrival of the first VBR outbreak at a municipality according to biogeographic region: Influence of significant biotic and abiotic conditions at the time of arrival of the first VBR outbreak at each municipality. Correlation between the time of the first outbreak (e.g. number of years since 2000) in a municipality and its cattle population (top). Each plot (dot shape) represents a biogeographical region; Correlation between the time of the first outbreak in a municipality and the geographical distance to that outbreak to the closest reporting office (bottom). Each plot represents estimates at the biogeographic region level. Lines represent the tendency estimated for the prediction of the model and the shadow represents the confidence interval (CI=95%, grey) estimated using the method 'loess' in the *geom_smooth* function in ggplot2 in R.
